# The Utility of Non-Technical Skills for Surgeons (NOTSS) as an Assessment Tool in Surgical Training

**DOI:** 10.7759/cureus.74676

**Published:** 2024-11-28

**Authors:** Adam Gittins

**Affiliations:** 1 Paediatric Surgery, Alder Hey Children's Hospital Trust, Liverpool, GBR

**Keywords:** acceptability, educational value, feasibility, medical education assessment, non-technical skills, surgery curriculum, surgical training, utility, validity and reliability

## Abstract

Research indicates that poor communication accounts for almost half of errors made during surgery. Despite this, formal training in the cognitive and interpersonal aspects of operating is lacking in the surgical curriculum. Non-Technical Skills for Surgeons (NOTSS) is a behaviour rating system designed to evaluate surgeons on their non-technical skills in the operating room. This rating system splits intraoperative non-technical skills into four domains: situation awareness, decision-making, communication and teamwork, and leadership. Assessors score surgical trainees on each of these domains, using these ratings to inform a structured debrief.

NOTSS has been shown to have a robust construct, content and concurrent validity, accurately predicting the risk of adverse events in surgery. Its reliability is threatened by considerable inter-rater variance, particularly in the cognitive domains of the taxonomy, which makes it difficult to defend its use as a summative assessment, but it is relatively procedure-independent as an assessment tool. The feasibility of incorporating NOTSS into the surgical curriculum depends on the viability and financial cost of training assessors across the United Kingdom (UK) in its use, which is currently unknown. It has been received positively by assessors and trainees alike as a formative tool to facilitate a deeper understanding of non-technical skills in theatre, but its acceptability as a barrier to progression has not been examined. Promisingly, a small study has demonstrated NOTSS assessments to have a positive impact on trainees’ non-technical proficiency in surgery, but further research is warranted before any definitive conclusions can be drawn about its educational value.

## Introduction and background

Non-Technical Skills for Surgeons (NOTSS) is a behaviour rating system developed in the United Kingdom (UK) for the assessment of surgeons’ non-technical proficiency in the operating room [1]. Non-technical skills can be defined as the cognitive and social skills that enable individuals and teams to accomplish their tasks [2]. Research indicates that poor communication is a contributing factor in as many as 43% of errors made during surgery [3]. An independent review in 2013 highlighted the need to implement training in non-technical skills into the surgical curriculum [4]. Despite this, surgical training continues to focus heavily on the technical aspects of operating, and formal instruction in non-technical skills is consequently often neglected.

NOTSS was created to address this lack of non-technical training in the surgical curriculum. It is the most widely used assessment of surgeons’ non-technical proficiency. The NOTSS rating system has earned acceptance from institutions such as the Royal College of Surgeons of Edinburgh (RCSEd) and the Royal Australasian College of Surgeons, but it is yet to gain recognition as a workplace-based assessment in the UK surgical curriculum. This article will evaluate the utility of NOTSS, considering its validity and reliability as an assessment tool, as well as the feasibility, acceptability and educational impact of its formal incorporation into surgical training [5].

## Review

Design and development

NOTSS was devised as a behaviour rating system to assess surgeons’ non-technical skills in the operating theatre. It was produced from the results of critical incident interviews with 27 consultant surgeons in Scotland [1], supplemented by a literature review. The surgeons were interviewed in relation to challenging operative cases from their experience, and cognitive cues were used to probe into the non-technical factors that impacted the event. Analysis of these interviews revealed four categories of non-technical skills in the operating room: situation awareness, decision-making, communication and teamwork, and leadership. The first two of these are considered cognitive categories, and the latter two are social categories. These four categories form the basis of the NOTSS taxonomy [6], shown in Table 1. Each of the four categories contains three elements, with examples of good and bad behaviours for each element. This three-tiered hierarchy mirrors behavioural rating systems that have been adopted in the field of anaesthetics [7] and the aviation industry [8].

**Table 1:** NOTSS taxonomy version 1.2 Adapted from NOTSS System Handbook version 1.2 [6] NOTSS: The Non-Technical Skills for Surgeons

Domain	Category	Element
Cognitive	Situation awareness	Gathering information
		Understanding information
		Projecting and anticipating future state
	Decision-making	Considering options
		Selecting and communicating option
		Implementing and reviewing decisions
Interpersonal	Communication and teamwork	Exchanging information
		Establishing a shared understanding
		Coordinating team activities
	Leadership	Setting and maintaining standards
		Supporting others
		Coping with pressure

The NOTSS rating form is comprised of a four-point Likert scale used by an assessor to mark the surgeon’s non-technical skills. The assessor observes the surgeon during a theatre case, scoring each element from one (poor performance) to four (good performance). An even-point scale was selected to eliminate a neutral option. The assessor is encouraged to write feedback on the candidate’s performance for each element. The four global categories are then assigned an overall score. Notes on the rating form are used to inform a structured debrief after the case consisting of feedback and discussion on performance.

The NOTSS rating system has been designed as a formative assessment tool for the purpose of training surgeons in the acquisition of non-technical skills. It is intended for use by senior surgeons in assessing their trainees, but the assessor could theoretically be any member of the theatre team, given its emphasis on the non-technical aspects of operating.

The NOTSS taxonomy was informed largely by the insights of 27 consultant surgeons across three surgical subspecialties (general, orthopaedic and cardiothoracic) working in Scotland. This may limit the generalisability of the tool to other subspecialties and countries. The study design seems to treat these consultants as experts in operative non-technical skills. However, no details are given on the non-technical skills training of these surgeons. The authors themselves even acknowledge that there has historically been a lack of formal education in non-technical skills in surgical training. As a result, this presumption of expertise may not be justified, as operative experience and technical proficiency do not necessarily translate to mastery of the non-technical aspects of operating. Nonetheless, the findings from these interviews were supplemented by an extensive review of the literature to generate the NOTSS taxonomy, which included data from questionnaires, other interviews, observational studies and adverse events analyses [9]. The development of NOTSS was therefore grounded in a robust evidence base.

Validity

The validity of NOTSS as an assessment tool rests on its ability to accurately measure a surgeon’s non-technical skills in theatre. At face value, NOTSS appears to capture all relevant non-technical skills, and the higher-order categories resemble those found in behavioural rating systems used for other members of the theatre team [7,10]. The systematic method of element selection in the development of the NOTSS taxonomy further supports its content validity. In a randomised controlled trial, medical students who received targeted training in non-technical skills performed better on NOTSS assessments than those who did not [11], which provides evidence for its construct validity. Confirmatory factor analysis has demonstrated that the four-category configuration of the NOTSS taxonomy is a superior fit compared to alternative models. The rating system has acceptable internal consistency, with Cronbach’s alpha score exceeding 0.7 for each of the four categories, indicating that the elements are suitably grouped within their respective categories [12]. These findings support the validity of the current arrangement of the NOTSS taxonomy as depicted in Table 1.

The rationale behind the development of NOTSS is that enhancing surgeons’ non-technical skills will ultimately result in fewer adverse events during surgery and improved patient safety. An observational study at the Royal Infirmary of Edinburgh demonstrated the relationship between poor non-technical skills and preventable intraoperative incidents [13]. In this study, poor behaviours observed using the NOTSS taxonomy were associated with a much higher frequency of avoidable incidents. Concurrent criterion validity testing has shown a positive correlation between increased NOTSS scores and higher patient safety scores as rated by consultant surgeons [12]. Research therefore indicates that NOTSS is a valid predictor of intraoperative incidents and patient safety in theatre.

As a workplace-based assessment, NOTSS has high validity according to Miller’s pyramid of clinical competence [14]. However, caution must be exercised in generalising a candidate’s performance in a workplace-based assessment to their clinical practice. If the trainee is aware that they are being assessed, they may modify their behaviours, compromising the validity of the NOTSS assessment as an accurate reflection of clinical performance. Assessors could mitigate this risk by not disclosing to trainees that they are undertaking a NOTSS assessment in advance of the theatre case, but this may have negative consequences for the acceptability of the assessment tool to trainees.

Reliability and sources of variance

Reliability refers to the consistency of a measuring tool. For an assessment, a reliability coefficient above 0.7 is generally considered acceptable [15], with a higher threshold of 0.8 sometimes adopted for high-stakes summative assessments [5]. Behaviour rating systems are particularly prone to subjective interpretation, which can threaten the reliability of the tool. Developers of the NOTSS system sought to evaluate its reliability by examining the inter-rater agreement between 44 surgeons, who were asked to observe six simulated operating scenarios and score the lead surgeon using the NOTSS rating system [16]. The study found that NOTSS had acceptable inter-rater reliability in the communication and teamwork (rWG=0.70) and leadership (rWG=0.72) categories of the NOTSS rating tool but considerable variability in the situation awareness (rWG=0.51) and decision-making (rWG=0.66) categories. This finding is consistent with participant feedback elsewhere in the literature, which has noted the difficulty in rating surgeons in the cognitive categories of the NOTSS taxonomy [17]. It is worth noting that most assessors in this study had no prior experience with NOTSS. Inter-rater reliability of NOTSS assessments can be improved with greater expertise and further training [18].

Variance component analysis can identify the factors contributing to the variation of scores in an assessment tool. In an ideal assessment, candidate ability would be the only source of variance. Indeed, non-technical proficiency makes the largest contribution to the NOTSS score, but only by a small margin. Assessor stringency and subjectivity are also significant contributing factors to NOTSS score variation, making the overall reliability of the NOTSS assessment lower than technical skills assessments used in theatre [19]. However, the NOTSS score is relatively procedure-independent compared to technical assessments. These findings suggest that NOTSS is more susceptible to assessor-based variance, but less susceptible to procedure-based variance, than assessments of operative technical proficiency. Increasing the number of examiners would reduce the effect of assessor-based variance and increase overall reliability, but research suggests that as many as 6-8 assessors would be required to attain a level of reliability sufficient for high-stakes summative assessments [17], which would be difficult to achieve in the workplace.

Feasibility and cost-effectiveness

For an assessment to be introduced on a wider scale, its implementation must be achievable and cost-effective. Incorporation of NOTSS into the surgical curriculum would require training of senior surgeons in its use to enable them to appropriately rate and provide feedback on the non-technical skills of their trainees. This training is of particular importance given the lack of formal education in non-technical skills that many consultant surgeons will have received in their training. The NOTSS Handbook recommends that each surgical department appoint a small number of trained NOTSS assessors, who should undergo training in human factors and the NOTSS rating system [6]. RCSEd has released free online NOTSS Introductory Videos, designed to provide an overview of the principles of the rating system [20]. However, ensuring the assessment is valid and reliable would likely require more extensive training. To properly equip senior surgeons in assessing trainees, RCSEd runs the NOTSS Masterclass, a one-day paid course that provides a more comprehensive education in the use of the system [20]. The capacity of these courses to train NOTSS assessors in every surgical department in the country, as well as the financial cost of such an endeavour, remains inconclusive.

The implementation of a new theatre-based assessment must not be overly time-consuming so as to cause disruption to the operating list. In this respect, NOTSS performs well when compared to similar workplace-based assessments. In a study comparing non-technical theatre-based assessments, novice assessors took an average of 10.1 minutes to complete a NOTSS form, which was faster than the assessment tools used for anaesthetists and scrub nurses [21]. The time taken to debrief varies by assessor but should take an average of 3-5 minutes [22]. The total time for a NOTSS assessment and debrief should therefore take no longer than around 15 minutes, which could comfortably fit in between theatre cases with little to no disruption.

The question of which member of the theatre team should act as NOTSS assessor has implications for the feasibility of the assessment. Many would argue that the consultant surgeon is the most appropriate given their experience in leading theatre cases and communicating with theatre staff as a surgeon, although this may not always be practical. If the consultant surgeon is required to assist in the operation, they cannot devote their full attention to observing the non-technical skills of the trainee. In such cases, the anaesthetist or operating department practitioner could perform the assessment, but they would not be able to draw from personal operative experience when giving feedback to the trainee, which may limit the quality of the learning.

There are notable threats to the feasibility of NOTSS assessments. As previously discussed, assessors have reported difficulty in rating trainees on the cognitive categories of the form [17]. This may be owing to the cognitive behaviours being by nature harder to observe without the operating surgeon articulating their thought processes. Additionally, evaluating a trainee in the leadership category requires them to take a leading role in the theatre case, which may prove challenging for more junior trainees, particularly in tertiary centres with a high volume of complex consultant-led cases. Such departments may struggle to provide enough opportunities for core surgical trainees to demonstrate their leadership qualities intraoperatively. If NOTSS assessments were to become a mandatory part of surgical training across all grades, departments would need to be confident they can provide sufficient theatre cases in which junior trainees can act as lead surgeons. Other logistical obstacles to completing a NOTSS assessment include last-minute schedule changes to the operating list and emergency situations forcing the NOTSS assessor to discontinue the assessment to focus on operating or other clinical priorities [17].

Acceptability

Response to the NOTSS assessment, both from assessors and those being assessed, has been largely positive. Most assessors agree that NOTSS provides a common language to discuss non-technical skills and is useful in supporting reflection. Only a minority feel its use adds too much time to the operating list [17]. Surgical trainees have responded that the debrief encourages deeper analysis of non-technical aspects of operating, and they find the feedback useful to inform their future practice [22,23]. However, participants in many studies investigating NOTSS were recruited through targeted advertising or involvement in previous studies, rather than through random selection. This may introduce a selection bias favouring assessors and trainees who have a prior interest in teaching or developing non-technical skills and whose views may not be representative of the wider cohort of eligible assessors and surgical trainees. It is also important to note that the positive reception to NOTSS as a formative assessment tool should not be extrapolated to its use as a summative assessment tool, given that the feedback from trainees pertains to its value as an educational device and not as a barrier to progression.

In developing a new assessment tool, care must be taken to ensure it does not unfairly discriminate against particular groups. In a study comparing the non-technical performance of surgical trainees, NOTSS scores displayed a positive correlation with years of UK training, but not with years of non-UK training [17]. Whilst there are many possible explanations for this discrepancy, it does pose a potential risk of differential attainment between surgeons trained in the UK and those trained in other parts of the world. Further research is required to establish whether such a difference in NOTSS performance truly exists and the factors contributing to this difference.

Educational impact

Assessments can be used to train and test the learner’s proficiency in the subject matter. In the case of NOTSS, an explicit purpose given by the authors is to develop surgeons’ non-technical skills in theatre, by identifying specific areas of improvement through a structured debrief with constructive feedback. This debrief process has been shown to be effective in developing trainees’ non-technical performance. An interventional study found that the average NOTSS score of surgical trainees improved significantly following a single debrief session [23]. Furthermore, all participants found the debrief to be beneficial to their education, with most agreeing that feedback on non-technical skills in the operating room should be incorporated into surgical training. Given the high validity of NOTSS as an assessment tool for measuring non-technical skills, this study provides promising evidence that serial NOTSS assessments with a structured debrief enhance intraoperative non-technical performance. However, this study only evaluated 11 participants; further research is warranted with larger sample sizes in order to confirm the educational benefit of NOTSS assessments.

There are features of the NOTSS assessment that may in fact impede learning. Like many other workplace-based assessments, NOTSS requires assessors to rate trainee performance on a numerical scale. Grading competence in this way has been shown to reduce students’ intrinsic motivation and therefore engagement with learning [24]. Moreover, the assignment of a numerical score may shift the trainee’s focus away from feedback and recommendations on their performance, hindering subsequent learning from the debrief. Removing grading from the NOTSS assessment may help to concentrate the focus of both trainer and trainee on the debrief, which is the central mechanism for development in this assessment.

## Conclusions

The importance of intraoperative non-technical skills in preventing adverse events in surgery is well-documented in the literature. However, surgical training has changed little in response to this research, and the curriculum is still lacking in formal education on these cognitive and interpersonal skills. NOTSS is a workplace-based assessment designed to support surgical trainees in their non-technical proficiency in theatre. It was generated from a robust evidence base and has been shown to have high validity in measuring surgeons’ intraoperative non-technical skills and predicting avoidable adverse incidents. Its reliability is below the threshold for high-stakes assessments and is threatened by assessor subjectivity and stringency. Inter-rater agreement can be improved with assessor training and experience, but the effect size of these measures on the reliability of the tool has not been investigated. From a feasibility viewpoint, NOTSS assessments can usually be completed in 15 minutes or less, without disruption to theatre lists. Assessor training courses are run throughout the year, but the cost and capacity of expanding these courses to facilitate nationwide NOTSS assessments as part of surgical training will require further investigation. NOTSS has found good acceptability by assessors and trainees alike, who have valued the opportunity it provides to discuss cognitive and interpersonal skills in the theatre environment. An interventional study has highlighted the positive educational value of NOTSS assessments, but this was with a small sample size.

In summary, NOTSS has been demonstrated to have high validity and acceptability as a formative assessment tool for surgical trainees, although its reliability is not sufficient to defend its use as a summative assessment. Large-scale studies into its feasibility and educational impact are required before NOTSS can be incorporated into the UK surgical training curriculum.
